# Case report: Immediate revascularization for symptomatic hepatic artery pseudoaneurysm after orthotopic liver transplantation? A case series and literature review

**DOI:** 10.3389/fsurg.2023.1169556

**Published:** 2023-06-27

**Authors:** An Verena Lerut, Jacques Pirenne, Mauricio Sainz-Barriga, Joris Blondeel, Geert Maleux, Diethard Monbaliu

**Affiliations:** ^1^ Department of Abdominal Transplant Surgery, University Hospitals Leuven, Leuven, Belgium; ^2^ Laboratory of Abdominal Transplantation, Department of Microbiology, Immunology and Transplantation, KU Leuven, Leuven, Belgium; ^3^ Department of Radiology, University Hospitals Leuven, Leuven, Belgium

**Keywords:** case series, hepatic artery pseudoaneurysm, orthotopic liver transplantation, endovascular treatment, arterial reconstruction

## Abstract

**Introduction:**

Hepatic artery pseudoaneurysm (HAPA), a rare vascular complication that can develop after liver transplantation, is associated with a high mortality rate and graft loss. To salvage the liver graft, immediate revascularization, either through surgical or endovascular intervention, is required. However, currently there is no consensus on the optimal strategy. Here, we report three cases of liver transplant recipients diagnosed with HAPA and treated with immediate revascularization. In addition, we present an overview of HAPA cases described in the literature and make recommendations on how to treat this rare complication.

**Methods:**

All adults transplanted in our center between 2005 and 2021 were retrospectively reviewed. Literature search was done in PubMed for original studies between 1980 and 2021 reporting early hepatic artery (pseudo) aneurysm after liver transplantation requiring either surgical or endovascular intervention.

**Results:**

From a total of 1,172, 3 liver transplant patients were identified with a symptomatic HAPA and treated with immediate revascularization. HAPA occurred 73, 27, and 8 days after liver transplantation and was treated with immediate revascularization (two surgical and one endovascular intervention). Literature review identified 127 cases of HAPA. HAPA was managed with endovascular therapy in 20 cases and by surgical intervention in 89 cases. Overall reported mortality rate was 39.6%, whereas overall graft survival was 45.2%.

**Conclusion:**

Immediate surgical or radiological interventional excision and prompt revascularization to salvage liver grafts is feasible but still associated with a high mortality.

## 1. Introduction

Hepatic artery (pseudo) aneurysm (HAPA) is a rare and potentially life-threatening complication following orthotopic liver transplantation where a locally advanced infectious process causes dilation of the arterial anastomosis. The reported incidence varies between 0.3% and 3% with mortality rates up to 80% (1). Risk factors for HAPA are bile leaks, Roux-en-Y hepaticojejunostomy, and primary sclerosing cholangitis since all these conditions are associated with biliary infection and/or contamination of the operative field (2). Diagnosis may be incidental or made as a result of symptoms varying from abdominal pain or fever, to gastrointestinal bleeding with or without hemorrhagic shock. To avoid graft loss and/or patient death, early detection and prompt treatment are essential. Both surgical and endovascular interventions are reported. Surgical options are either hepatic artery ligation followed by early retransplantation, or HAPA resection followed by immediate revascularization with an interposition graft. Endovascular treatment can include stenting to restore vascularization or embolization. Currently, there is no consensus on the optimal treatment strategy and studies are scarce.

Here, we report three cases where HAPA was treated with immediate revascularization—surgical in two cases and endovascular in one case—and provide a review of current literature. As there is no accepted algorithm for the treatment of HAPA, we also make recommendations on how to treat this rare complication.

## 2. Case series

### 2.1. Case 1

**Figure 1:**
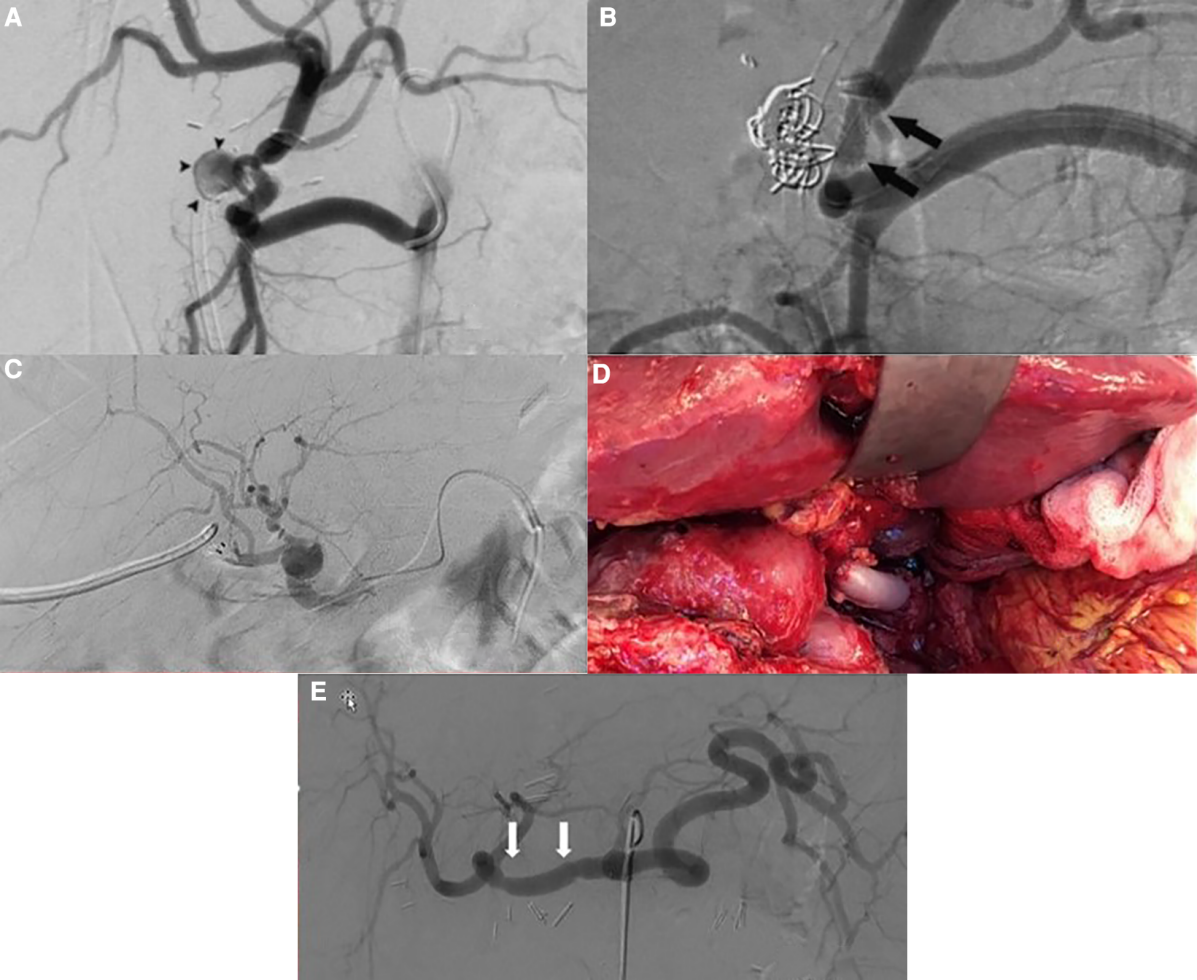
(**A**) Angiography showing the hepatic pseudoaneurysm; arrowheads point to pseudoaneurysm (case 1). (**B**) Angiography post coiling and stenting of the hepatic pseudoaneurysm; arrows point to the stent (case 1). (**C**) Post-LT selective angiography revealing an extrahepatic pseudoaneurysm with a preserved arterialization of the liver allograft (case 2). (**D**) Intraoperative view showing status after excision of the pseudoaneurysm and vascular reconstruction using a free iliac interposition graft between common hepatic and hepatic artery (case 3). (**E**) Angiography after repair of the hepatic artery pseudoaneurysm; arrows point to the proximal and distal anastomosis (case 3).

A 69-year-old woman with primary biliary cirrhosis and hepatorenal syndrome had undergone a combined liver and kidney transplantation. Patient characteristics can be found in Table 1. Seventy-three days after transplantation, she presented with a severe upper gastrointestinal bleeding resulting in hemorrhagic shock (Table 1). Following resuscitation measures, a percutaneous angiography revealed a bleeding HAPA, arising from the arterial anastomosis between the donor celiac artery and the recipient proper hepatic artery. Bleeding was immediately treated with coil embolization of the HAPA (Figure 1A). Despite hemorrhagic shock-related coronary insufficiency, the patient recovered well. Unfortunately, after 3 weeks, she was readmitted with severe gastrointestinal bleeding. Angiography confirmed recurrent bleeding from the HAPA. Considering the patient’s age, hemodynamic status, recent myocardial infarction, and the rapid recurrence, we opted for an endovascular approach where an expandable, covered stent was positioned at the level of the HAPA, thereby excluding the HAPA while preserving allograft arterialization (Figure 1B). Patency was confirmed at 6 months by CT and at 9 months MR angiography, and at 20 months by Doppler ultrasound. At the time of writing, almost 2 years after endovascular HAPA treatment, the patient is asymptomatic with normal liver tests and normal hepatic vascular parameters.

**Table 1:** Summary of characteristics of hospital course for each case.

Characteristics	Case 1	Case 2	Case 3
Recipient characteristics			
Age (years)	69	68	49
Gender	Female	Male	Male
Indication for LT	Primary biliary cirrhosis	Retransplantation	Retransplantation
		Ischemic type biliary strictures with cholangitis	Primary Sclerosing Cholangitis
MELD score	NI	26	12
Surgical procedure			
Cold ischemia time	NI	511	430
Warm ischemia time	NI	41	41
Arterial reconstruction			
Biliary reconstruction	PHA-TC	CHA-CHA	CHA-CHA/GDA
Postoperative course	Duct-to-duct	Duct-to-duct	Roux-Y hepatic-jejunostomy
Immunosuppression	Corticosteroids	Corticosteroids	Corticosteroids
	Mycophenolate mofetil	Mycophenolate mofetil	Mycophenolate mofetil
	Tacrolimus	Tacrolimus	Tacrolimus
Infection present	No	Yes	Yes
		*Staphylococcus epidermidis* and *Enterococcus faecium*	*Candida glabrata*
Biliary complications	Yes, anastomotic stricture on duct-to-duct biliary anastomosis	No	No
Acute anemia	Yes	Yes	Yes
Hypotension	Yes	Yes	Yes
Clavien–Dindo ≥3	Yes, IIIb, IV	Yes, IIIa, IV,V	Yes, IV
Days between surgery and diagnosis	73	27	8
Days from hypotensive/acute anemia episode to diagnosis	0	14	0
Diagnostic modality	Angiogram in acute setting	Angiogram	Emergency laparotomy
Therapeutic modality	Embolization followed by stenting	Excision of aneurysm and surgical reconstruction	Excision of aneurysm and surgical reconstruction
Mortality	No (follow-up >20 months)	Yes (39 days)	No (follow-up >24 months)

LT, liver transplantation; NI, no information; PHA, proper hepatic artery; TC, celiac trunk; CHA, common hepatic artery; GDA, gastroduodenal artery; MELD, model of end stage liver disease.

### 2.2. Case 2

**Figure 2:**
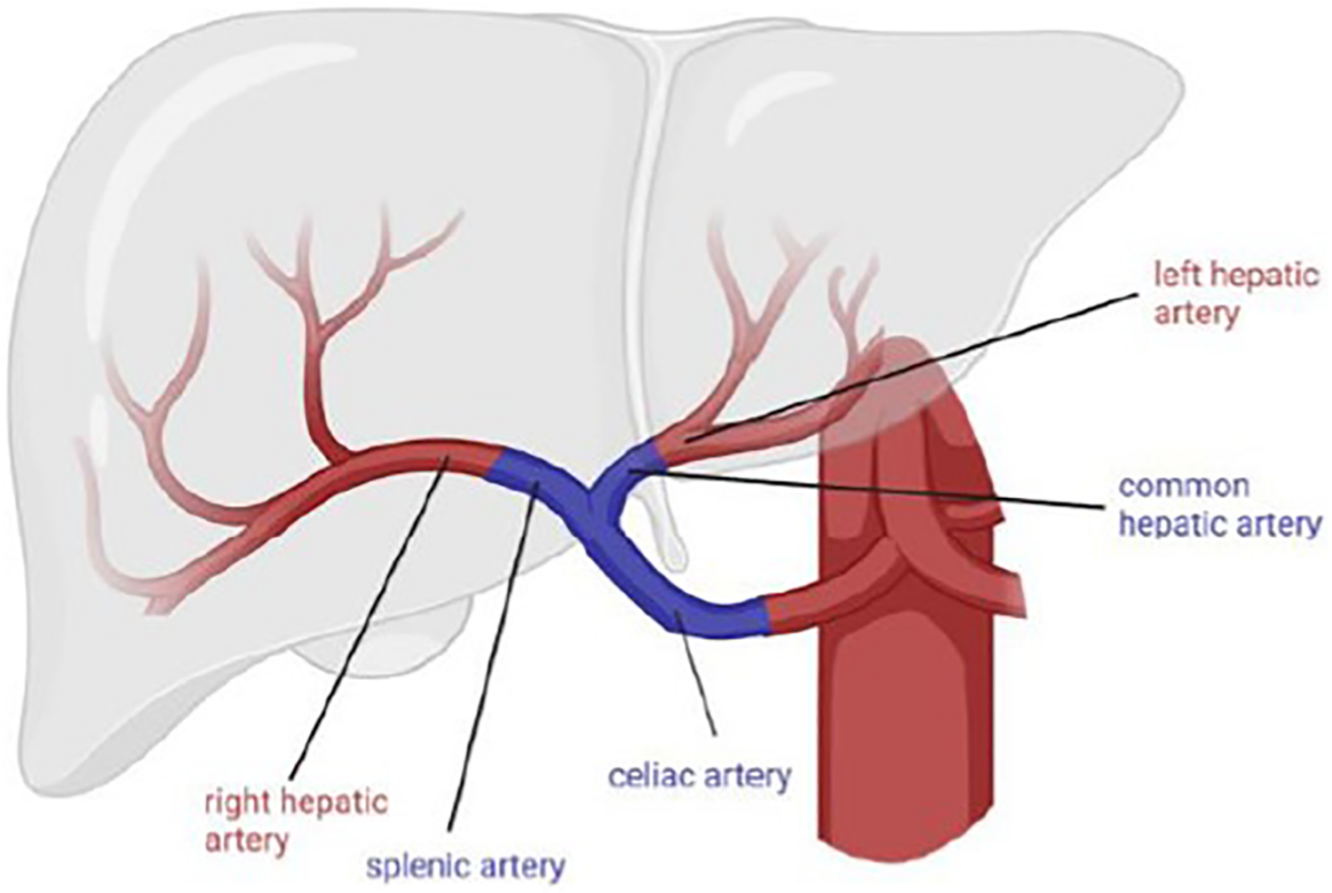
Arterial reconstruction as described in case 2.

A 68-year-old male was retransplanted for recurrent cholangitis related to ischemic cholangiopathy. Patient characteristics can be found in Table 1. Perioperative bile cast cultures were positive for *Staphylococcus epidermidis*, whereas *Enterococcus faecium* was cultured postoperatively from the abdominal drains. On postoperative day 14, the patient developed a fever up to 38.9°C, for which antibiotic therapy with vancomycin was initiated. The next day, he passed melena stool and biochemistry showed a hemoglobin drop (Table 1). Endoscopy revealed a duodenal ulcer, for which pantoprazole was initiated. Doppler ultrasound on day 16 showed turbulent flows in a tortuous hepatic artery, indicative of arterial stenosis. The patient developed acute abdominal pain and a CT scan revealed a confined perforation of the duodenal ulcer, which was treated conservatively with nasogastric tube aspiration and nil per mouth policy. Antibiotic therapy was escalated with meropenem and fluconazole in addition to vancomycin. One day later he developed a neutropenic fever with several hypotensive episodes responding to fluid challenges. On day 24, a *de novo* atrial fibrillation occurred and was managed pharmacologically. Meanwhile, his abdominal pain worsened, and an intra-abdominal collection was identified and drained percutaneously from which *Enterococcus faecium* was cultured. On day 27, he developed hematochezia (melena and anal red blood loss) leading to a hemoglobin drop (6 g/dl) despite transfusion with 4 units of packed red blood cells. Angiography did not demonstrate any active bleeding but—in retrospect—showed a saccular HAPA (Figure 1C) but unfortunately then interpreted as a wide arterial anastomosis. Two days later, he was urgently transferred to the operating theater with acute hemorrhagic shock, where a bleeding HAPA was found. The HAPA was resected, and immediate revascularization was established using an arterial allograft consisting of the celiac artery with the splenic artery and common hepatic artery. The splenic artery and common hepatic artery were anastomosed to the right and left hepatic arteries of the allograft, respectively, and the celiac artery was anastomosed to the recipients’ common hepatic artery (Figure 2). Unfortunately, Doppler ultrasound the day after showed a diffuse necrosis of the allograft in the absence of a normal arterial flow and the patient died at day 39 due to sepsis and multiorgan failure.

### 2.3. Case 3

A 49-year-old male was retransplanted for recurrent primary sclerosing cholangitis with biliary cirrhosis and portal vein thrombosis. Prior to retransplantation, he had been hospitalized for 3 months for recurrent severe esophageal variceal bleedings, refractory cholangitis, and recurrent bacteriemia, with need for continuous intravenous antibiotic treatment with meropenem. Patient characteristics are shown in Table 1. Eight days after transplantation, he suddenly developed a life-threatening hemorrhagic shock with massive blood loss via the abdominal drains (Table 1). At emergency laparotomy, a bleeding HAPA was identified. The aneurysm was resected and the allograft re-arterialized using a 3 cm iliac arterial interposition graft from the same donor restoring continuity between the proper hepatic artery and common hepatic artery (Figure 1D). Because a fungal infection was suspected, fluconazole was added to the anti-infectious regimen. Cultures of the aneurysm later confirmed the presence of *Candida glabrata*. Accordingly, the dose of fluconazole was doubled. As perioperative cultures were also positive for *Lactobacillus* species, meropenem was continued for another 2 weeks. Hereafter, his recovery was uneventful with repeated normal arterial blood flow on Doppler ultrasound. Twelve days after re-arterialization, a *Clostridium difficile* infection was treated with a 10-day course of oral vancomycin. Control angio-CT showed patent intra- and extrahepatic arterial vasculature (Figure 1E). At the time of writing, 1 year later, the patient is asymptomatic with normal liver tests and normal hepatic vascular parameters.

## 3. Literature review

Literature search was done in PubMed for studies between 1980 and 2021. All original studies reporting early (within 3 months after liver transplantation) hepatic artery (pseudo) aneurysm after liver transplantation requiring either surgical or endovascular intervention were included. In total, 127 cases were identified over a period of 40 years in 28 different centers. All studies are presented in Table 2, and the overall results are summarized in Table 3.

**Table 2:** Overview of hepatic artery (pseudoaneurysms after liver transplantation published between 1988 and 2022).

Author Ref Year	Pat Nr	Age	G	Ind PSC	Culture	Symptom	Delay LT-D (days-mean), days	Biliary reconstruction	Bile leak Nr	Arterial reconstruction	HAPA site	R/	Treatment modality Nr	Mortality	PS Nr	GS Nr
Houssin (3) 1988	2	18 33	F 2	0	*Enterobacter cloacae* (*n* = 2)	GIB (*n* = 1) HS (*n* = 1)	33 72	RYHJ (*n* = 1) DD (*n* = 1)	0	PHA-CHA PHA-CTR	Anas	S	Lig 2	1/2	A 1 (1 year) D 1 (110 days)	1
Lerut (4) 1988	4	NR	NR	NR	NR	HS (*n* = 4)	NR	NR	NR	NR	Anas (*n* = 2)	S	Exc + revasc 1 None 3	3/4	A 1 D 3	1
Langnas (5) 1991	5	NR	NR	0	*Pseudomonas aeruginosa* (*n* = 2) *Staphylococcus aureus* (*n* = 1) *Candida albicans* (*n* = 2)	GIB (*n* = 2) HS (*n* = 3)	13.4 (5–27)	RYHJ (*n* = 1) DD (*n* = 4)	NR	NR	NR	S	Exc + revasc 3 Exc + *graft* Revasc 2	1/5	A 4 D 1: – 1× Exc + graft	4
Madariaga (6) 1992	7	42 (5–58)	F 4 M 3	1	*Enterococcus species* (*n* = 1) *Streptococcus* (*n* = 1) *Candida species* (*n* = 1)	GIB (*n* = 4) HS (*n* = 1)	47 (10–70)	RYHJ (*n* = 2)	4	Aortic jump graft (*n* = 3)	NR	IR S	Emb 1 Lig 6	3/7	A 4 (2–5 years) D 3 – 1× Emb – 2× Lig (15–117 days)	6
Soin (7) 1995	1	47	M 1	0	No infection	INC	90	DD	1	HAbif-CTR LHA-SPLA	Anas	S	Exc + graft revasc 1	0	A (9 months)	1
Fichelle (8) 1997	1	30	M 1	0	*Staphylococcus aureus*	HS	30	RYHJ	0	CHA-CTR	Anas	S	Exc + *graft* revasc 1	0	A (5 years)	1
Lowell (9) 1999	2	37, 52	M2	0	No infection	GIB (*n* = 1) INC (*n* = 1)	NR	RYHJ (*n* = 1)	0	NR	NR	S	Exc + revasc 1 Exc + *graft* Revasc 1	0	A 2 (21–24 mo)	2
Stange (10) Settmacher (11) 2000	5	45 (31–65)	M 5	2	*Proteus species* (*n* = 1) *Staphylococcus species* (*n* = 2) *Candida species* (*n* = 2)	GIB (*n* = 2) HS (*n* = 1) INC (*n* = 1) Card dec (*n* = 1)	120.5 (28–300)	CHOL DUOD STOMY (*n* = 2) DD (*n* = 3)	NR	PHA-GDA 3 PHA-SPLA 2	Anas (*n* = 3) CTR (*n* = 1) Dist ANAS (*n* = 1)	S	Lig 3 Exc + *graft* revasc 2	2/5	A 3 D 2: – 2× Exc + graft (6 months, 3 years)	5
Leonardi (12) 2001	1	30	M	0	NR	GIB	119	NR	1	CHA-CHA	Anas (*n* = 1)	S	Exc *graft* revasc 1	0	A 24 m (HAT graft reLT)	0
Marshall (13) 2001	9	43 (13–62)	NR	5	*Candida species* (*n* = 3) *Aspergillus species* (*n* = 1)	GIB (*n* = 2) HS (*n* = 6) Fever (*n* = 1)	32.8 (8–75)	RYHJ (*n* = 7)	4	Aortic jump graft 4	NR	IR S	Emb 3 Lig 3 Exc + Revasc 1 Exc + *graft* revasc 1 ReLT 4 (post-Emb 3×/post-LIG 1×) None° 1	2/9	A 2 D 7: – 3× Lig; 1× RLT – 1× Emb – 1× Exc + revasc – 1× Exc + graft – 1× None	0
Almogy (14) 2002	1	45	M1	1	*Citrobacter freundii*	GIB	31	RYHJ	0	NR	NR	IR	Emb 1	0	A	1
Turrion (15) 2002	4	NR	NR	NR	NR	HS (*n* = 4)	NR	NR	NR	NR	NR		None° 4	4/4	D 4	0
Leelaumdonlipi (16) 2003	8	36.5 (32–59)	NR	NR	*Streptococcus species* (*n* = 1) *Gemella haemolysans* (*n* = 1) *Streptoc faecalis* (*n* = 1) *Enterococcus species* (*n* = 1) *Haemophilus paraproph* (*n* = 1) *Candida species* (*n* = 2) *Aspergillus species* (*n* = 1)	GIB (*n* = 2) HTens (*n* = 4) Abnrl LT (*n* = 2)	122.2 (12–760)	RYHJ (*n* = 5)	NR	CTR-CHA 2 CHA-CHA 2 CHA_GDA 1 CHA_RHA 1 SA -CHA 1	NR	S	Exc + revasc 2 Exc + *graft* revasc 6	4/8	A 4 (8.6–12.8 years) D 4: – 1× Exc + revasc – 3× Exc + graft	4
Patel (17) 2003	1	70	F 1	0	NR	GIB	50	DD	1	CHA-CHA/GDA	Anas	IR	Emb 1(thrombin)	0	A (6 mo)	1
Alamo (18) 2005	2	43 2 pt NR	M 2 pt NR	NR	*Pseudomonas* (*n* = 2) *Enterobacter* (*n* = 2)	HS (*n* = 2)	NR	NR	NR	NR	NR	S	Exc + revasc 1 Tx-ectomy 1	½	A 1 D 1: – 1× Tx-ectomy	1
Finley (19) 2005	2	46,55	F 1 M 1	0	*Aspergillus fumigatus* (*n* = 1)	GIB (*n* = 2)	26 7 years	NR	1	NR	Anas (*n* = 2)	S	Exc + *graft* revasc 2	0	A 2 (16–72 months)	2
Kim (20) 2005 LDLT	11	52 (43–65)	F 2 M 9	0	*Candida species* (*n* = 1)	HTens (*n* = 5) Abnrl LTests (*n* = 1) INC (*n* = 5)	13.8 (6–40)	NR	NR	PHA-PHA	Anas (*n* = 7) Stump HA (*n* = 1) CHA (*n* = 2) RGA (*n* = 1)	NR	NR	NR	NR	NR
Fistouris (21) 2006	11	NR	NR	NR	*Enterococcus faecium* (*n* = 1) *Klebsiella pneumoniae*. (*n* = 2) *Enterococcus species* (*n* = 1) *Candida albicans* (*n* = 5) *Candida glabrata* (*n* = 1)	GIB (*n* = 6) HTens (*n* = 4) Pain (*n* = 2) Anemia (*n* = 1) Abdominal bleed (*n* = 1)	67.5 (14–240)	RYHJ (*n* = 6)	3	NR	NR	IR S	Lig 6 Exc + *graft* revasc 3 ReLT 5 (post-Lig 4×, post revasc 1×) None° 2	6/11	A 5 (7.4 months–6.9 years) D 6: – 2× Lig – 1× Exc + graft – 2× None	1
Jain (22) 2006	4	52 (40–65)	F 3 M 1	0	NR	NR	174.75 (1–482)	NR	NR	NR	NR	IR S	Emb 1 Revasc + Lig 1 Exc + *graft* revasc 1 ReLT 1 (post revasc + LIG) None° 1	1/4	A 3 (141 months) D 1: – None	3
Saad (23) 2011	9	52 (46–63)	F 2 M 7	NR	NR	GIB (*n* = 3) HS (*n* = 2) HTens (*n* = 5)	49.5 (1–196)	RYHJ (*n* = 5) DD (*n* = 4)	NR	NR	Anas (*n* = 7) RHA (*n* = 1) CHA (*n* = 1)	IR	Stent 3 Exc + revasc 1 ReLT 4 (post stent 1×) None° 1	2/9	A 7 D 2: – 1× Stent – 1× None	2
Volpin (2) 2014 REVIEW	16	40 (20–62)	F 6 M 10	1	*Enterococcus species* (*n* = 2) *Staphylococcus aureus* (*n* = 2) *Methicillin resistant staphylococcus aureus* (*n* = 2) *Pyocyaneus*/*G. bacilli* (*n* = 2) *Pseudomonas aeruginosa*/*E. coli* (*n* = 1) *Aspergillus fum* (*n* = 1)	GIB (*n* = 3) HS (*n* = 13) Abdominal bleed (*n* = 5)	25.1 (4–100)	RYHJ (*n* = 6) DD (*n* = 10)	4	CTR-PHA 4 CTR-CHA 1 SPLA-AO1 PHA-PHA 3 CHA-CHA 2 RHA-RHA 4 CHA-GRAFT-CHA 1	NR	IR S	Emb 1 Stent 1 Lig 5 Exc + revasc 5 Exc + *graft* revasc 2 None °2	8/16	A 8 (34 months–18 years) D 8: – 3× Lig – 1× Exc + revasc – 1× Exc + graft – 2× None	7
Reznichenko (24) 2016	1	56	F 1	0	NR	Pain	180	DD	0	CHA-CHA	Anas	S	Exc + revasc 1	0	A (4 months)	1
Jeng (25) 2016 REVIEW	2	44 63	M 2	0	NR	HS (*n* = 2)	38 147	NR	0	NR	Anas (*n* = 1) RHA (*n* = 1)	IR S	Emb1 Exc + Revasc 2	2/2	D 2	0
Harrison (26) 2017 REVIEW	7	49 (28–62)	F 2 M 5	2	*Klebsiella pneumoniae* (*n* = 4) *Pseudomoas aeruginosa* (*n* = 1) *Candida caselifl* (*n* = 1)	NR	46 (4–133)	RYHJ (*n* = 3) DD (*n* = 4)	3	CHA-CHA 5 CHA-PHA 1 CHA-graft AO	NR	NR	NR	2/7	A 5 D 2	5
Mohkam (27) 2017	3	52 (45–62)	NR	0	NR	NR	68 (29–147)	NR	NR	CTR-CHA	Anas (*n* = 1)	IR	Stent > Revasc 1 Exc + *graft* revasc 3	0	A 3 (3.5, 269 months)	3
Gao (28) 2019	1	59	M 1	0	NR	Jaundice	NR	NR	NR	NR	NR	IR	Stent 1	0	A	1
St. Michel (29) 2019	4	52 (37–62)	F 1 M 3	0	*Enterococcus species* (*n* = 1) *Stenotropohm maltoph* (*n* = 2)	HTens (*n* = 2) anemia (*n* = 4)	49.2 (15–84)	NR	1	NR	NR	IR S	Emb 2 Exc + revasc 2	2/4	A 1 (24 months) D 2: – 2× Emb	2
Present 2023	3	62 (49–69)	F 1 M 2	1	*Enterococcus faec* (*n* = 1) *E. coli* (*n* = 1) *Candida glab*. (*n* = 1)	GIB (*n* = 1) HS (*n* = 1) Abdominal bleed (*n* = 1)	36.6 (8–79)	RYHJ (*n* = 1)	1	CHA-CHA/GDA 3	Anas (*n* = 3)	IR S	Emb 1 + Stent 1 Exc + *graft* revasc 2	1/3	A 2 (20.24 mo) D 1	2

CR, case report; CS, case series; GIB, gastrointestinal bleed; HS, hemorrhagic shock; HTens, hypotension; INC, incidental find; RYHJ, Roux-en-Y Hepaticojejunostomy; DD, duct to duct anastomosis; PHA, proper hepatic artery; CHA, common hepatic artery; CTR, celiac trunk; LHA, left hepatic artery; SPLA, splenic artery; GDA, gastroduodenal artery; IR, interventional radiology; Emb, embolization; Lig,, ligation; Exc + revasc, excision and revascularization; Exc + graft revasc, excision and graft revascularization; PS, patient survival; GS, graft survival; PSC, primary sclerosing cholangitis; MRSA, methicillin resistant staphylococcus aeureus; Card dec, cardiac decompensation; Abnrl LT, abnormal liverfunction test; Chol, choledochus; Duod stomy, duodenostomy; Anas, anastomosis; HAbif, hepatic arteriy bifurcation; RHA, right hepatic artery.

Refs. (10) and (11) similar series.

**Table 3:** Summary of all reported cases.

Total of reported cases	*N* = 127
Patient characteristics	
Age, years (Median, IQR)	50 (38–62)
Gender	Female *n* = 26
	Male *n* = 56
Indication	
PSC	*N* = 13 (10.2%)
Re-LT	*N* = 7 (5.5%)
Symptomatology	
Gastrointestinal bleed	*N* = 32 (25.2%)
Hemorrhagic shock	*N* = 41 (32.3%)
Abdominal bleed	*N* = 7 (5.5%)
Hypotension	*N* = 20 (15.7%)
Pain	*N* = 3 (2.4%)
Abnormal liver function test	*N* = 3 (2.4%)
Incidental finding	*N* = 8 (6.3%)
Not reported	*N* = 13 (10.2%)
Days to diagnosis (mean, days)	57.9 (1–760)
Biliary reconstruction	
Roux-en-Y	*N* = 40 (31.5%)
Infection	
Bacterial	*N* = 44 (34.6%)
Fungal	*N* = 22 (17.3%)
No infection	*N* = 2 (1.6%)
Not reported	*N* = 59 (46.5%)
Therapeutic modality	
Interventional radiology	
Embolization	*N* = 13 (10.2%)
Stenting	*N* = 7 (5.5%)
Surgery	
Ligation	*N* = 25 (19.7%)
Excision + revascularization	*N* = 21 (16.5%)
Excision + graft revascularization	*N* = 28 (22%)
Transplantectomy	*N* = 1 (0.8%)
Retransplantation	*N* = 14 (11%)
None	*N* = 14 (11%)
Not reported	*N* = 18 (14.2%)
Mortality	*N* = 50/126 (39.6%)
Reported patient survival (*N* = 116)	*N* = 63 (54.3%)
Reported graft survival (*N* = 126)	*N* = 57 (45.2%)

PSC, primary sclerosing cholangitis; Re-LT, re liver transplantation.

Clinical presentation of HAPA varied widely. Detailed information about symptomatology was reported in 114 cases. Thirty-two percent of reported patients presented with a hemorrhagic shock, whereas other presentations included gastrointestinal bleeding (i.e., melena and hematemesis) (*n* = 32, 25%), hypotension (*n* = 20, 15.7%), intra-abdominal bleeding (*n* = 7, 5.5%), pain (*n* = 3, 2.4%), or abnormal liver function tests (*n* = 3, 2.4%). Mean time between transplantation and diagnosis is 58 days (range 1–760). In 84 cases, a risk factor associated with HAPA was present: primary sclerosing cholangitis (*n* = 13), Roux-en-Y hepaticojejunostomy (*n* = 40), bile leak (*n* = 24), or retransplantation (*n* = 7). Out of the 127 cases, perioperative cultures were reported in 68 cases, revealing 44 bacterial (34.6%), 22 fungal infections (17.3%), and 2 negative cultures (1.6%). No detailed information on the treatment was given in 18 cases. Endovascular treatment was done in 20 cases: stenting in 7 and embolization in 13. Surgical intervention was performed in 76 cases including ligation (*n* = 25, 23.8%), excision and revascularization (*n* = 21, 16.5%), excision and revascularization with graft (*n* = 28, 22%), and transplantectomy (*n* = 1, 0.8%). Unfortunately, in 13 (13%) of all the cases, no treatment could be offered. Fourteen patients received a retransplantation (12.8%). Overall mortality in the literature is 39.6% with a graft survival of 45.2%.

## 4. Discussion

Hepatic arterial thrombosis and hepatic artery aneurysms are the most feared complications after liver transplantation. They occur in up to 4% and 2%–3% respectively (2, 13, 16). In case of liver transplantation, hepatic artery aneurysm is also referred to as hepatic artery pseudoaneurysm or HAPA. Hepatic artery aneurysm formation is due to arterial wall dissection or infection (26). In 1885, the term mycotic aneurysm was first mentioned by Osler but nowadays infected aneurysm is the preferred term as it is known that a “mycotic aneurysm” can also be caused by bacterial pathogens (30). Infected aneurysms are more common in immune-compromised patients such as diabetic, oncologic, and transplant patients (18). Diagnosis of a hepatic artery aneurysm may be incidental at imaging or made as a result of symptoms varying from vague abdominal pain syndrome, anemia, obstructive jaundice, or fever, to severe life-threatening conditions caused by massive gastrointestinal or intra-abdominal bleeding (31–33).

HAPA most commonly occurs within the first 3 months as in case 2 and 3. In case of late (after 3 months) presentation as in our first case, it is hypothesized that the HAPA is not associated with an infection. Patients who present late are usually asymptomatic (incidental finding) or have insidious abdominal complaints (34). As shown in Table 3, an overall graft loss of 54.8% and an overall patient mortality of 46.7% are reported. Our small case series including one endovascular stenting and two excisions with immediate graft revascularization reports a similar graft and patient survival of 66%.

Since clinical presentation varies widely, early diagnosis relies on a high index of suspicion. Reported risk factors include biliary leakage, biliodigestive anastomosis (mostly Roux-Y hepatic-jejunostomy), and primary sclerosing cholangitis. Indeed, all these conditions are associated with biliary infection or contamination of the operative field (2, 10, 26). As shown in Table 3, in 31.5% of all reported cases, biliary reconstruction was performed by Roux-Y hepaticojejunostomy, whereas in 10.2%, indication for liver transplantation was primary sclerosing cholangitis. The presence of these risk factors in patients who present with a post-transplant gastrointestinal or abdominal bleeding should therefore alert the clinician to consider a possible HAPA.

Up to date, there is no clear diagnostic algorithm for HAPA. The literature review as well as our small case series clearly highlight the importance of a rapid diagnosis but even more of an urgent treatment. Indeed, early detection of a HAPA after liver transplantation has been shown to contribute to improved survival (25). Routine Doppler ultrasound has been shown to improve the detection of vascular complications after liver transplantation (35). Visualization of a hilar cystic structure abutting the vessels and presenting a turbulent, bidirectional, or slow monophasic flow should immediately raise suspicion for a HAPA. Multidetector angio-CT or MR are both suggested to confirm the diagnosis. Additional angiography could be considered as this allows to locate more precisely the site of the hepatic artery aneurysm as well as to judge the arterialization of the allograft. Moreover, angiography offers, as shown in case 1, the possibility to intervene during the same procedure.

Despite awareness, the diagnosis of HAPA often remains difficult. In the series of Kim et al., the median time between living donor liver transplantation and diagnosis of HAPA was 10 days; routine imaging using Doppler ultrasound and CT scan allowed us to detect HAPA in 6 out of 11 patients (54%). The Seoul group showed, when comparing different imaging methods, that angio-CT and multidetector CT are the imaging modality of choice to diagnose HAPA (20). Nevertheless, in our series (case 2), the diagnosis of HAPA on angio-CT was initially missed, which delayed timely treatment.

Equally, there is no consensus on optimal treatment strategies. Here, lessons from larger experiences in infected aortic surgery can be learned. The two guiding principles are, first, the immediate start of antibiotic therapy in case infection is present at endovascular or open surgery in order to minimize the risk of prosthetic graft contamination by hematogenous spread. Second, a surgical repair is preferred unless the perioperative risk is prohibitively high (cf. case 1 in our series). Surgical treatment should include debridement with complete excision of the infected vessel or graft, followed—whenever possible—by an *in situ* reconstruction using free vascular auto- or allografts. If unavailable, an extra-anatomic reconstruction could be opted for using a prosthetic graft impregnated with antibiotics. Endovascular arterial repair is considered a bridge to a more definite treatment or, in the absence of gross contamination, a viable option for patients at risk or unfit for open surgery (36). Endovascular repair entails an infectious risk as the infected aneurysmal sac and surrounding tissues remain. The latter being the reason why endovascular aortic repair for mycotic aortic aneurysm is still debated despite the extensive experience with endovascular stent repair in abdominal aortic aneurysm. Endovascular aortic repair is indeed associated with a poor prognosis when pre- and postoperative blood cultures are positive and lifelong oral antibiotic therapy is required (37).

Although the etiology of HAPA compared to an infected aorta or visceral artery seems totally different, the fundamental principles of endovascular repair may well be adapted to effectively treat HAPA following liver transplantation. Antibiotics should be administered during a period of 6–8 weeks if inflammatory signs are present at the time of treatment. This therapy must be adapted and eventually prolonged and guided by results of blood and tissue cultures (38). As shown in Table 3, a specific pathogen was identified in 34.6% of the reported cases and a mycosis was present in 17.3% of them.

The treatment of HAPA after liver transplantation should be guided by (i) the clinical condition of the patient, (ii) the vascularization of the allograft, and (iii) finally the location of the aneurysm. Although an endovascular approach is without doubt an attractive solution to a difficult problem, one should keep in mind that the HAPA is frequently infected in the transplant setting (28, 39). An intraluminal stent may, therefore, act as an infectious nidus. Saad et al., therefore, propose to use stents as a temporizing measure to prevent (further) bleeding while preserving the arterial flow to the allograft. This measure allows the transplant team to evaluate the salvageability of the graft and consider later on surgical revascularization or retransplantation (23). Endovascular treatment may also be preferable in the case of late appearance of hepatic aneurysms (cf. case 1 in our series). Moreover, these patients frequently present with some arterial collateralization, allowing a more aggressive exclusion of the aneurysm.

When an endovascular repair is not possible or unsuccessful, urgent laparotomy is often indicated. Depending on the intraoperative findings, there are two surgical options: ligation of the feeding arteries or excision of the aneurysm followed by an arterial reconstruction. Such reconstructions should preferably be done using donor arterial allografts, if possible from the same donor. If a “donor vascular toolkit” is not available, autologous saphenous, iliac, and left renal veins, or cryopreserved arterial allografts can be used. These allografts can be safely used after a storage period reaching up to 2 weeks despite destruction of the endothelium. Larger experiences have shown that arterial allografts are superior to prosthetic grafts and more physiologic compared to venous allografts (40). In living donor liver transplantation, cryopreserved iliac artery allografts have been frequently used successfully. Wang et al. showed that there is no loss of patency in cryopreserved allografts, which have been stored for more than 1 year (41). Mohkam et al. and Sellers et al. corroborated these findings in deceased donor liver transplantation cases requiring urgent arterial revascularization (27, 42). If a patient suffers from extensive local infection or significant hemodynamic instability, the only potential life-saving measures usually involve ligating the artery to control bleeding, removing the transplanted organ due to anticipated allograft necrosis, and creating a temporary portocaval shunt. These interventions should be accompanied by long-term, comprehensive anti-infectious treatment and urgent relisting for a subsequent retransplantation (4). In some cases of late HAPA formation, extensive arterial collateralization may allow us to keep the allograft “alive” despite ligation of the hepatic artery.

We recognize the limitations of our study. Inherent to the low incidence of HAPA, the study design was retrospective and mostly covered small case reports and series with incomplete reporting of data and a possible focus on unusual and/or successful cases. Because of the nature of reporting, data on actual follow-up times are not available. Nevertheless, we believe that our study brings together valuable insights into the diagnosis and treatment of HAPA.

## 5. Conclusion

HAPA is a rare but life-threatening complication after liver transplantation. A high index of suspicion during the early post-transplant period, especially in case of unexplained hemoglobin drop or symptomatic gastrointestinal/abdominal bleeding, is paramount to salvage both the liver graft and the patient. Physicians should be extra vigilant in case of any previous biliary leakage, biliodigestive reconstruction, or primary sclerosing cholangitis as the indication for transplantation. Routine Doppler ultrasound and especially angio-CT scan are the best diagnostic procedures to diagnose HAPA. In a hemodynamically (un)stable patient, endovascular repair may be a valid therapeutic option to bridge patients for definite surgical treatment and/or retransplantation, or even as a definite treatment in late presenting cases preferably without evidence of infection. However, in case of hemorrhagic shock, urgent surgical exploration is usually required, where, if feasible, excision of the HAPA followed by a re-arterialization using vascular allografts is the preferred therapeutic option. Evidently, any treatment modality requires stringent radiological follow-up to assess vascular parameters, as well as graft perfusion.

## Data Availability

The raw data supporting the conclusions of this article will be made available by the authors, without undue reservation.

## References

[1] Piardi T Lhuaire M Bruno O Memeo R Pessaux P Kianmanesh R Vascular complications following liver transplantation: a literature review of advances in 2015. World J Hepatol. (2016) 8:36–57. 10.4254/wjh.v8.i1.36 26783420 PMC4705452

[2] Volpin E Pessaux P Sauvanet A Sibert A Kianmanesh R Durand F Preservation of arterial vascularization after hepatic artery pseudoaneurysm following orthotopic liver transplantation: long-term results. Ann Transplant. (2014) 19:346–52. 10.12659/AOT.890473 25034853

[3] Houssin D Ortega D Richardson A Ozier Y Stephan H Soffer M Mycotic aneurysm of the hepatic artery complicating human liver transplantation. Transplantation. (1988) 46:469–72. 10.1097/00007890-198809000-00033 3047941

[4] Lerut JP Gordon RD Tzakis AG Stieber AC Iwatsuki S Starzl TE. The hepatic artery in orthotopic liver transplantation. Helv Chir Acta. (1988) 55:367–78. 3049463 PMC3086426

[5] Langnas A Wagner M Stratta RJ Wood P Shaw BW. Early complications after orthotopic liver transplantation. Am J Surg. (1991) 161:76–83. 10.1016/S0039-6109(05)70009-8 1987861

[6] Madariaga J Tzakis A Zajko AB Tzoracoleftherakis E Tepetes K Gordon R Hepatic artery pseudoaneurysm ligation after orthotopic liver transplantation-a report of 7 cases. Transplantation. (1992) 54:824–8. 10.1097/00007890-199211000-00011 1440848 PMC3154769

[7] Soin AS Jamieson NV. Native hepatic artery pseudoaneursym after liver transplantation: an unusual presentation with biliary leak. Eur J Vasc Endovasc Surg. (1995) 10:376–9. 10.1016/S1078-5884(05)80063-2 7552545

[8] Fichelle JM Colacchio G Castaing D Bismuth H. Infected false hepatic artery aneurysm after orthotopic liver transplantation treated by resection and reno-hepatic vein graft. Ann Vasc Surg. (1997) 11:300–3. 10.1007/s100169900050 9140607

[9] Lowell JA Coopersmith CM Shenoy S Howard TK. Unusual presentations of nonmycotic hepatic artery pseudoaneurysms after liver transplantation. Liver Transpl Surg. (1999) 5:200–3. 10.1002/lt.500050306 10226110

[10] Stange B Settmacher U Glanemann M Nuessler NC Bechstein WO Neuhaus P. Aneurysms of the hepatic artery after liver transplantation. Transplant Proc. (2000) 32:533–4. 10.1016/S0041-1345(00)00877-0 10812100

[11] Settmacher U Stange B Haase R Heise M Steinmüller T Bechstein WO Arterial complications after liver transplantation. Transpl Int. (2000) 13:372–8. 10.1016/B978-0-7216-0118-2.50068-9 11052274

[12] Leonardi LS Soares C Boin IFSF Oliveira VC. Hemobilia after mycotic hepatic artery pseudoaneurysm after liver transplantation. Transplant Proc. (2001) 33:2580–2. 10.1016/S0041-1345(01)02103-0 11406253

[13] Marshall MM Muiesan P Srinivasan P Kane PA Rela M Heaton ND Hepatic artery pseudoaneurysms following liver transplantation: incidence, presenting features and management. Clin Radiol. (2001) 56:579–87. 10.1053/crad.2001.0650 11446757

[14] Almogy G Bloom A Verstandig A Eid A. Hepatic artery pseudoaneurysm after liver transplantation. Transpl Int. (2002) 15:53–5. 10.1007/s00147-001-0373-x 11875615

[15] Turrion VS Alvira LG Jimenez M Lucena JL Ardaiz J. Vascular complications in a series of 300 orthotopic liver transplants. Transplant Proc. (2002) 34:292–3. 10.1016/S0041-1345(99)00406-6 11959290

[16] Leelaudomlipi S Bramhall SR Gunson BK Candinas D Buckels JAC McMaster P Hepatic-artery aneurysm in adult liver transplantation. Transpl Int. (2003) 16:257–61. 10.1111/j.1432-2277.2003.tb00296.x 12730806

[17] Patel J V Weston MJ Kessel DO Prasad R Toogood GJ Robertson I. Hepatic artery pseudoaneurysm after liver transplantation: treatment with percutaneous thrombin injection. Transplantation. (2003) 75:1755–7. 10.1097/01.TP.0000063936.94587.10 12777870

[18] Álamo JM Gómez MA Tamayo MJ Socas M Valera Z Robles JA Mycotic pseudoaneurysms after liver transplantation. Transplant Proc. (2005) 37:1512–4. 10.1016/j.transproceed.2005.02.046 15866659

[19] Finley DS Hinojosa MW Paya M Imagawa DK. Hepatic artery pseudoaneurysm: a report of seven cases and a review of the literature. Surg Today. (2005) 35:543–7. 10.1007/s00595-005-2987-6 15976950

[20] Kim HJ Kim KW Kim AY Kim TK Byun JH Won HJ Hepatic artery pseudoaneurysms in adult living-donor liver transplantation: efficacy of CT and Doppler sonography. Am J Roentgenol. (2005) 184:1549–55. 10.2214/ajr.184.5.01841549 15855114

[21] Fistouris J Herlenius G Bäckman L Olausson M Rizell M Mjörnstedt L Pseudoaneurysm of the hepatic artery following liver transplantation. Transplant Proc. (2006) 38:2679–82. 10.1016/j.transproceed.2006.07.028 17098038

[22] Jain A Costa G Marsh W Fontes P Devera M Mazariegos G Thrombotic and nonthrombotic hepatic artery complications in adults and children following primary liver transplantation with long-term follow-up in 1000 consecutive patients. Transpl Int. (2006) 19:27–37. 10.1111/j.1432-2277.2005.00224.x 16359374

[23] Saad WEA Dasgupta N Lippert AJ Turba UC Davies MG Kumer S Extrahepatic pseudoaneurysms and ruptures of the hepatic artery in liver transplant recipients: endovascular management and a new iatrogenic etiology. Cardiovasc Intervent Radiol. (2013) 36:118–27. 10.1007/s00270-012-0408-y 22648698

[24] Reznichenko AA Bondoc A Paterno F Shah SA. Hepatic artery pseudoaneurysm after liver transplantation. J Gastrointest Surg. (2016) 20:1405–6. 10.1007/s11605-016-3097-z 26839001

[25] Jeng KS Huang CC Lin CK Lin CC Liang CC Chung CS Early detection of a hepatic artery pseudoaneurysm after liver transplantation is the determinant of survival. Transplant Proc. (2016) 48:1149–55. 10.1016/j.transproceed.2015.11.017 27320576

[26] Harrison J Harrison M Doria C. Hepatic artery pseudoaneurysm following orthotopic liver transplantation: increasing clinical suspicion for a rare but lethal pathology. Ann Transplant. (2017) 22:417–24. 10.12659/AOT.903367 28684726 PMC12577485

[27] Mohkam K Darnis B Rode A Hetsch N Balbo G Bourgeot JP Rescue arterial revascularization using cryopreserved iliac artery allograft in liver transplant patients. Exp Clin Transplant. (2017) 15:420–4. 10.6002/ect.2016.0116 28350292

[28] Gao W Li X Huang L. Treatment of obstructive jaundice caused by hepatic artery pseudoaneurysm after liver transplantation: a case report. Medicine (Baltimore). (2019) 98:23–6. 10.1097/MD.0000000000018015 PMC6940052 31860951

[29] St. Michel DP Goussous N Orr NL Barth RN Gray SH LaMattina JC Hepatic artery pseudoaneurysm in the liver transplant recipient: a case series. Case Rep Transplant. (2019) 2019:1–6. 10.1155/2019/9108903 PMC6959152 31976118

[30] Osler W. The Gulstonian lectures, on malignant endocarditis. Br Med J. (1885) 1:522–6. 10.1136/bmj.1.1263.522 20751196 PMC2255907

[31] Abbas MA Fowl RJ Stone WM Panneton JM Oldenburg WA Bower TC Hepatic artery aneurysm: factors that predict complications. J Vasc Surg. (2003) 38:41–5. 10.1016/S0741-5214(03)00090-9 12844087

[32] Rosenberg A Trebska-McGowan K Reichman T Sharma A Cotterell A Strife B Management of hepatic artery aneurysm: a case series. Ann Hepatobiliary Pancreat Surg. (2020) 24:333–8. 10.14701/ahbps.2020.24.3.333 32843601 PMC7452805

[33] Hosn MA Xu J Sharafuddin M Corson JD. Visceral artery aneurysms: decision making and treatment options in the new era of minimally invasive and endovascular surgery. Int J Angiol. (2019) 28:11–6. 10.1055/s-0038-1676958 30880885 PMC6417896

[34] Oderich GS Panneton JM Bower TC Cherry KJ Rowland CM Noel AA Infected aortic aneurysms: aggressive presentation, complicated early outcome, but durable results. J Vasc Surg. (2001) 34:900–8. 10.1067/mva.2001.118084 11700493

[35] Singh AK Nachiappan AC Verma HA Uppot RN Blake MA Saini S Postoperative imaging in liver transplantation: what radiologists should know. Radiographics. (2010) 30:339–51. 10.1148/rg.302095124 20228321

[36] Lee WK Mossop PJ Little AF Fitt GJ Vrazas JI Hoang JK Infected (mycotic) aneurysms: spectrum of imaging appearances and management. Radiographics. (2008) 28:1853–68. 10.1148/rg.287085054 19001644

[37] Stenson KM Grima MJ Loftus IM Tripathi RK. Recommendations for management of infected aortic pathology based on current evidence. Semin Vasc Surg. (2019) 32:68–72. 10.1053/j.semvascsurg.2019.07.003 31540659

[38] McCready RA Bryant MA Divelbiss JL Chess BA Chitwood RW Paget DS. Arterial infections in the new millenium: an old problem revisited. Ann Vasc Surg. (2006) 20:590–5. 10.1007/s10016-006-9107-y 17039259

[39] Luo CM Chan CY Chen YS Wang SS Chi NH Wu IH. Long-term outcome of endovascular treatment for mycotic aortic aneurysm. Eur J Vasc Endovasc Surg. (2017) 54:464–71. 10.1016/j.ejvs.2017.07.004 28826996

[40] Martínez JA Rigamonti W Rahier J Gigi J Lerut J De Ville de Goyet J Preserved vascular homograft for revascularization of pediatric liver transplant. Transplantation. (1999) 68:672–7. 10.1097/00007890-199909150-00013 10507487

[41] Wang C Lopez-Valdes S Lin T-L Yap A Yong C-C Li W-F Outcomes of long storage times for cryopreserved vascular grafts in outflow reconstruction in living donor liver transplantation. Liver Transpl. (2014) 20:173–81. 10.1002/lt 24382821

[42] Sellers MT Haustein S V McGuire BM Jones C Bynon JS Diethelm AG Use of preserved vascular homografts in liver transplantation: hepatic artery aneurysms and other complications. Am J Transplant. (2002) 2:471–5. 10.1034/j.1600-6143.2002.20513.x 12123215

